# Supplementary material for: Complexome profiling of the Chlamydomonas psb28 mutant reveals TEF5 as an early PSII assembly factor
Source: Plant Cell. 2025 Mar 17;37(6):koaf055. doi: 10.1093/plcell/koaf055 (PMC12164590; doi:10.1093/plcell/koaf055)
Supplement: koaf055_Supplementary_Data [file koaf055_supplementary_data.zip › psb28_interactive.zip!/plots/intensity/qualityPlots/peptides/FAP186_Cre03.g156250.t1.2.html]

### Cre03.g156250.t1.2

Trivial name: FAP186  
SeqCoverage [median]: 24.40  
Mass: 22.76 kDa  
  
 

| Sequence | MissedCleavage | Mass [Da] | wtIntensity | muIntensity | Score |
| --- | --- | --- | --- | --- | --- |
| EATLLYNQLK | 0 | 1191.65 | 4046.41 | 24167.40 | 146.81 |
| GLLTMLTNK | 0 | 989.56 | 0.00 | 1802.96 | 85.06 |
| IGLDFGVSMGDKVDK | 1 | 1579.79 | 0.00 | 6868.90 | 78.93 |
| NAAVNTQTAQTDAASGAK | 0 | 1717.82 | 1416.12 | 2963.95 | 171.67 |
| VDKNAAVNTQTAQTDAASGAK | 1 | 2060.01 | 0.00 | 1260.06 | 59.81 |
|  |  |  | 5462.5 | 37063.3 |  |
